# The effect of myofascial therapy on postpartum rectus abdominis separation, low back and leg pain, pelvic floor dysfunction: A systematic review and meta-analysis

**DOI:** 10.1097/MD.0000000000035761

**Published:** 2023-11-03

**Authors:** Yueting Wang, Shuang Zhang, Peiqiang Peng, Wenxi He, Haitao Zhang, Haiyan Xu, Hong Liu

**Affiliations:** a School of Nursing, Jilin University, Chaoyang District, Changchun, P. R. China; b Department of Rehabilitation Medicine, Jilin Province FaW General Hospital - Lvyuan Branch, Changchun, P. R. China; c Department of Rehabilitation Medicine, Pan Shi Hospital, Jilin, P.R. China; d Department of Rehabilitation Medicine, The Second Hospital of Jilin University, Nanguan District, Changchun, P. R. China.

**Keywords:** ability to perform activities of daily living, common postpartum problems, myofascial therapy, pain

## Abstract

**Background:**

During pregnancy and postpartum, changes in biomechanics can cause dysfunctions in the myofascial system, such as rectus abdominis diastasis, various types of pain, and pelvic floor dysfunction. These common postpartum problems seriously threaten women’s health. Myofascial therapy, as an effective means of improving biomechanics, has no unified understanding of its therapeutic effects on postpartum functional disorders. This study aims to systematically evaluate the rehabilitative effects of myofascial therapy on postpartum rectus abdominis diastasis, low back and leg pain, and pelvic floor dysfunction through a meta-analysis of published randomized controlled trials.

**Methods:**

A systematic literature search of databases in Chinese and English was performed through May 2023. The treatment methods were randomized controlled studies using myofascial therapy in the treatment of rectus abdominis separation, lumbo-leg pain, and pelvic floor dysfunction. The main outcome indicators were abdominal circumference, rectus abdominis separation distance, visual analogue pain score, pelvic floor muscle potential, ability to live daily activities, number of events, and treatment effectiveness.

**Results:**

There were 22 studies, including 2235 patients. The result showed that compared with control group, myofascial therapy demonstrated to reduce abdominal circumference and rectus abdominis separation index, improve lumbar function significantly, and decrease urinary incontinence and pelvic organ prolapse. In the myofascial therapy group, pelvic floor muscle strength was significantly enhanced, anterior/posterior resting potential of pelvic floor muscle was significantly decreased, and pelvic floor muscle potential was enhanced. Compared with the control group, the number of patients with various types of pain and pain scores were significantly reduced after myofascial therapy. When myofascial therapy lasted <4 weeks, pain relief was greater. In the myofascial therapy group, the ability to perform daily activities was significantly improved. An analysis of the effectiveness of the treatment showed that after myofascial therapy, the patient’s symptoms improved significantly. There also saw low heterogeneity among all outcomes.

**Conclusion:**

The results suggested that myofascial therapy could effectively reduce rectus abdominis separation, relieve pelvic floor muscle dysfunction, enhance lumbar function, relieve pain, and improve the ability of daily living activities. All the data demonstrated that myofascial therapy had a good therapeutic effect on postpartum dysfunction.

## 1. Introduction

A number of postpartum disorders, such as rectus abdominis separation, abdominal circumference enlargement, pelvic leaning forward, lumbago, sacroiliac joint disorders, sacral pain, pelvic floor muscles weakness, urinary incontinence, pelvic organ prolapse, etc, are brought on by pregnancy, childbirth, and lack of rest after delivery. These conditions cannot be recovered naturally after delivery. Over time, women’s physical and mental health suffers due to impacts on significant muscle weakness, pain, which severely limited daily life abilities.

The main causes of these injuries are disturbance of the myofascial system caused by weight and hormonal changes. During pregnancy, the center of the body leans forward to balance the increase in the weight of the abdomen, coupling with the pelvis leaning forward, the pubococcygeal bone leaning backward, and the center of gravity of the body moving backward. This causes an excessive increase in the curvature of the lumbar spine, as well as an increase in the physiological curvature of the cervical and thoracic vertebrae. The body also produces forward tension as a result of increasing mammary gland bulk, shoulder blades and glenohumeral joints migrate inward toward the chest, and kyphosis or other aberrant postures develop. Further lordosis occurs in the lower abdomen as a result of the spine adjusting to reduce pressure on the lower abdomen.^[1,2]^ Long-term bad posture exacerbates biomechanical problems, including skeletal displacement to accommodate for upright walking, continual tension in the cervical, thoracic, lumbar, and abdominal muscles, and a restricted range of motion in the flexion, extension, and lateral flexion of the thoracolumbar region. On the other hand, during pregnancy, the increased progesterone destroyed the pelvic floor’s collagen fibers, resulting in decreased muscle strength for loss of protection of pelvic floor muscles and pelvic floor muscles elongated excessively.^[3]^ During pregnancy, the secretion of relaxin is also increased to aid in labor. Relaxin can weaken the pelvic floor support tissue by degrading collagen,^[1]^ which causes laxity in the ligaments surrounding the pubic symphysis and the sacroiliac joint, increases the spacing between the pubic symphysis. Laxity in the lumbar and abdominal muscles worsens pelvic pressure and puts additional strain on the pelvic floor muscles.^[4]^ Birth injuries, disturbance of core stabilizing mechanisms, insufficient abdominal pressure, and scarring are all possible during labor in both vaginal and cesarean deliveries.^[5]^ If there is insufficient rest after childbirth and excessive physical activity earlier, the body’s structure and function will be further harmed, leading to postpartum strain. The myofascial system may become over drafted as a result of these injuries. Therefore, myofascial system abnormalities can occur before, during, or after pregnancy. It will eventually cause rectus abdominis separation, dysfunction of the pelvic floor muscles, injuries to the lumbosacral and sacroiliac joints and ligaments, and injuries to the abdominal, lumbosacral, and sacroiliac muscles. Once injury occurs during the postpartum period, it is challenging on self-healing.

Myofascial chain is a tensile, integrated structure that includes ligaments, myofascial membrane, and is supported by bone. Through the chain network, each component works together to ensure the stability of the body’s structure and functionality. As an uninterrupted connective tissue, fascia has the characteristics of continuity, integrity, conduction and so on, and has the function of affecting the whole body. There is synergy between muscles and fascia to form larger interconnected anatomical chains.^[6]^ According to the myofascial chain theory, the myofascial membrane serves as the body’s conduction and support system for tension, and the functional state of the local myofascial membrane directly affects the overall myofascial chain.^[7]^ Therefore, once the local function is abnormal, the function of the corresponding remote part will be affected along the direction of stress conduction. In order to maintain overall balance, the other tissues of the body compensate for the tension deficit in the damaged tissue, further exacerbating the dysfunction of the entire fascial chain. It will help in the treatment of dysfunction by releasing or activating local fascia, inactivating myofascial pain triggers to relieve fatigue and pain in the body, and remodeling myofascial tension. Currently, the commonly used fascia therapy methods include myofascial manipulation,^[8]^ myofascial pain point therapy,^[9]^ extracorporeal shock wave therapy,^[10]^ massage manipulation,^[11]^ acupoint application,^[11]^ myofascial CC point,^[12]^ visceral myofascial manipulation,^[13]^ acupuncture,^[14]^ shockwave therapy,^[15]^ etc.

At present, some studies have shown that myofascial therapy was effective in musculoskeletal diseases, but there was still a lack of effective on the effect of common problems in postpartum women. The purpose of this study was to integrate the results of relevant studies and conduct a meta-analysis to clarify the influence of myofascial therapy on postpartum rectus separation, lumbago and pelvic floor muscle dysfunction, so as to provide a basis for clinical treatment.

## 2. Methods

The systematic review and meta-analysis were performed according to the preferred reporting items, no ethical approval and patient consent were required. The protocol number that we registered on the PROSPERO database is CRD42022376124.

### 2.1. Search strategy

Literature research included all studies on the treatment of postpartum rectus abdominis separation, pelvic floor dysfunction, and pelvic dysfunction with fascia therapy from the establishment of the database until May 5, 2023. Literature research was conducted independently by a researcher. First, titles and abstracts were selected according to inclusion and exclusion criteria. When the titles were unclear, the full text was reviewed. The following full text reading was done by 2 researchers. Differences should be resolved through discussion and agreement should be reached. We used the following keywords (using free text and MeSH search):

(((((Myofascial manipulation) OR (myofascial pain point therapy)) OR (Massage manipulation)) OR (CC point)) OR (viscera fascial manipulation).

(postpartum).

((((((((((((((Pelvic dislocation) OR (pubic symphysis separation)) OR (lower back pain)) OR (nonspecific low back pain)) OR (lumbago)) OR (lumbar instability)) OR (pelvic forward)) OR (sacroiliac joint disorder)) OR (tail bone pain)) OR (tailbone dislocation)) OR (sacroiliac joint pain)).

(((Rectus abdominis muscle separation) OR (waist circumference enlargement)) OR (abdominal subcutaneous fat thickening))).

(((((((((Pelvic misalignment) OR (sexual dysfunction)) OR (urinary incontinence)) OR (chronic pelvic pain)) OR (pelvic floor muscle dysfunction)) OR (fecal incontinence)) OR (constipation)) OR (dysuria)) OR (pelvic floor muscle weakness))).

Systematic searches of databases in Chinese (China Science and Technology Journal Database, Wanfang Data, and the China National Knowledge Infrastructure) and English (PubMed, Cochrane, Web of Science, Springer, and Elsevier) were performed for articles published from database inception until May 5, 2023. (Supplemental Digital Content, http://links.lww.com/MD/K468).

### 2.2. Study selection

At the earliest stages of our systematic review, rigid inclusion criteria were used to select relevant studies.

#### 2.2.1. Inclusion criteria

1. Study design: The study must be a randomized controlled study in English or Chinese, not an animal study.
2. Subjects: All subjects were patients with clinically confirmed Postpartum DRA or LBP or pelvic floor muscle dysfunction.
3. Intervention measures: The treatment group was given myofascial release therapy, and the intervention time was not limited, while the control group was given non-myofascial release therapy.

#### 2.2..2. Excluded criteria

1. Literature that was reported repeatedly.
2. Non-RCT studies.
3. Control studies of non-myofascial release therapy.
4. Abstracts, lectures, cases and reviews.
5. Studies in which the subject was not human.

### 2.3. Quality assessment

We assessed the methodological quality using the Cochrane Collaboration’s tool for assessing risk in Review Manager 5.3.^[16]^ There were 7 items provided by the tool to evaluate selection bias, performance bias, detection bias, attrition bias, reporting bias, and other biases. They included random sequence generation, allocation concealment, blinding of participants and personnel, blinding of outcome assessment, incomplete outcome data, selective reporting, and other biases. To assess the bias, each item was answered with 1 of 3 replies: “low risk,” “unclear risk,” and “high risk.”^[16]^

### 2.4. Data extraction

Two reviewers (YT W and H L) independently extracted the following information from each included study:

1. Basic information about the study, including the title, first author’s name, and publication date.
2. Characteristics of participants, including study population, sample size, age, number of pregnancies, mode of delivery, length of gestation, postpartum days, type of dysfunction, and treatment methods;
3. Main measurement indicators, including abdominal circumference, rectus abdominis separation index, visual analogue scale (VAS), electromyography of pelvic floor, activity of daily living (ADL), Low Back Pain Rating Scale (such as Japanese Orthopaedic Association scores (JOA) and Oswestry disability index (ODI) ect), pelvic Floor muscle Dysfunction Assessment Scale, and treatment effectiveness.

Any disagreement would be resolved by discussion between the 2 reviewers or consultation with the third reviewer (S Z).

### 2.5. Statistical analysis

RevMan 5.3 and Stata 15.0 were used to perform a meta-analysis. The weighted mean difference (WMD) or standardized mean difference indicated the quantitative influence of numerical or continuous factors, whereas the relative risk represented the effect size of binary variables. A 95% confidence interval (CI) was used to express each effect value. The CI was used to determine whether heterogeneity existed, and *I^2^* was used to monitor the extent of heterogeneity. Non-heterogeneity was considered present if *P* > .1 and *I^2^* < 40%; in this case, fixed effects models were employed in the meta-analysis. The source of heterogeneity was investigated if *P* < .1 and *I^2^* > 40%. Subgroup analyses were conducted according to the different study designs, study quality levels, and population characteristics when clinical or methodological heterogeneity existed between studies. A random-effects model was used when there was statistical heterogeneity between the studies but no clinical heterogeneity. If the factors contributing to heterogeneity were accurately measured and fully explained, a meta-regression analysis was performed. No meta-analysis was used, and only a general qualitative description of the results was provided when the level of heterogeneity was too high (*I^2^* > 40%), particularly when there was significant clinical or methodological heterogeneity and/or the source of heterogeneity could not be addressed by other methods. Subgroup analysis, funnel plot, and sensitivity analysis could be performed to find the cause of bias.

## 3. Results

### 3.1. Search result

**Figure 1.**
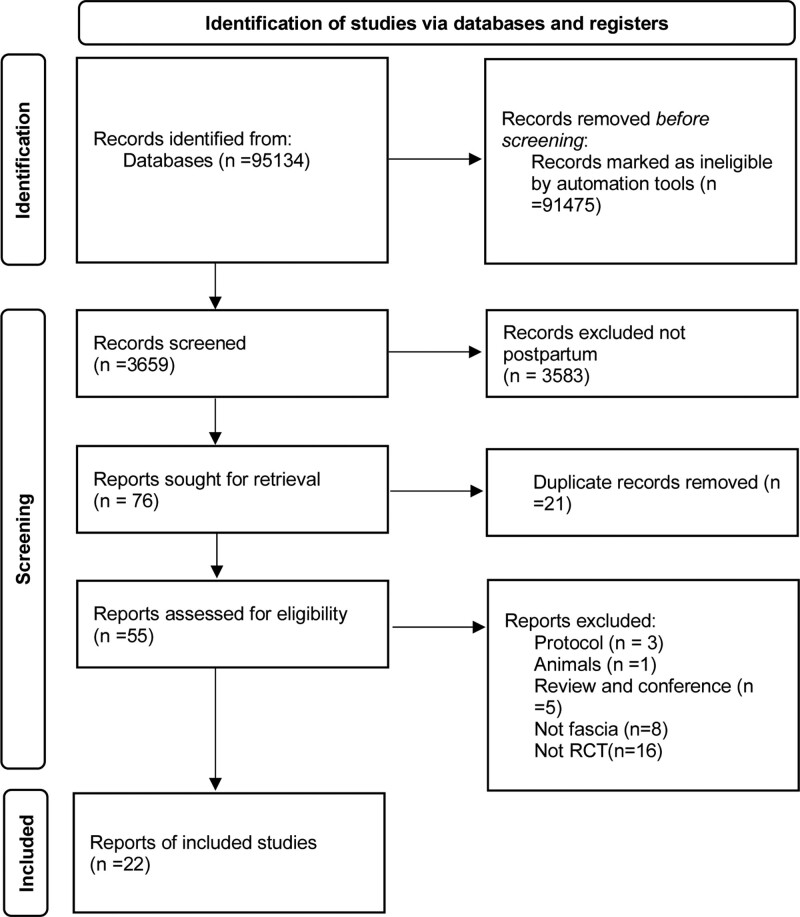
PRISMA flow diagram.

A total of 95,134 articles on myofascial therapy were identified through a literature search. After limiting keywords, removing duplications, animals research, experimental protocols, review/conference papers, and non-RCT articles, 22 studies were included in the final analysis (Fig. 1). There were 7 literatures on rectus abdominis,^[17–23]^ 5 literatures on pelvic/pubic symphysis separation,^[24–28]^ and 10 literatures on pelvic floor muscle.^[9,13,29–36]^ A total of 2235 patients were included in the analysis (Table 1). In our meta-analysis, baseline characteristics were comparable between myofascial therapy and control groups (Table S1, Supplemental Digital Content, http://links.lww.com/MD/K469).

**Table 1:** Summary of the studies.

Study	Year	Number		Main research topic	The control group	The intervention group	Type of experiment	Outcomes
		E	C					
QZ Xie	2022	31	31	Rectus abdominis muscle separation	CR + ES	ES + MM	RCT	①②③④⑧
JF Huang	2021	48	48	Rectus abdominis muscle separation	CR	ES + MM	RCT	①②
GY Zhou	2020	50	50	Pelvic floor rehabilitation	CR	ES + MM	RCT	④⑥⑦
WJ Zhang	2022	40	40	Rectus abdominis muscle separation	ES + ST	ES + ST + MM	RCT	②⑨
TT Fu	2020	35	35	Pelvic floor muscle fascia pain	ST	ST + MM	RCT	④⑨⑩
ZH Li	2023	51	51	Symphysis pubis dissociation	CR	MM	RCT	⑤⑥⑨⑩
Q Qiu	2021	38	38	Rectus abdominis muscle separation	CR + ES	ES + MM	RCT	②③⑨
Y Tao	2022	225	224	Pelvic floor rehabilitation	ES + MS	ES + MS + MM	RCT	⑨
BD Liao	2018	50	50	Rectus abdominis muscle separation	CR	ES + MM	RCT	①⑨
LJ Tian	2021	40	40	Weakness of pelvic floor muscles	CR	MM + ES	RCT	④
YM Chen	2022	50	50	Pelvic pain	ES	ES + FM fascia	RCT	⑦⑧⑨⑩
LI An	2019	100	70	Pelvic floor dysfunction	ES	ES + MM	RCT	④⑤⑥⑩
DF OY	2021	65	65	Rectus abdominis muscle separation	ES	ES + MM	RCT	①②⑤⑨
Ye Dong	2021	30	30	Stress urinary incontinence	ES	MM	RCT	④⑥
CF Zhou	2020	102	0	Pelvic floor muscle fascia pain	-	ES + MM	Before-after study	④⑩
LJ Luo	2022	30	30	Tension of pelvic floor muscles	ES	ES + MM	RCT	④⑩
YH Liu	2022	100	100	Rectus abdominis muscle separation	ES	ES + MM	RCT	②⑨⑩
XB Huang	2021	100	100	Pelvic pain and LBP	ST	ST + MM	RCT	⑤⑧⑩
J Cheng	2022	42	44	Pelvic pain	MM	MM + Myofascial acupuncture	RCT	⑤⑥⑩
ZT He	2020	30	30	Symphysis pubis dissociation	Belly bandit	MM + belly bandit	RCT	⑤⑩
XF Yang	2020	29	29	Pelvic floor muscle fascia pain	ES	ES + MM	RCT	④⑥⑩
María ÁG	2022	27	27	uracratia	CR + ES	MM	RCT	⑥

① Abdominal circumference; ② Rectus abdominis Separation Index; ③ Abdominal muscle strength and Rectus abdominis thickness; ④ Pelvic floor muscle Strength and vaginal pressure; ⑤ Pelvic dysfunction (Japanese Orthopaedic Association scores, JOA; Oswestry disability index, ODI); ⑥ Pelvic floor dysfunction (Female Sexual Function Index [FSFI]; urinary incontinence; Organ prolapse); ⑦ Sexual satisfaction or Female Sexual Function Index (FSFI) and nursing satisfaction; ⑧ ADL activities of daily living, ADL; ⑨ Therapeutic effectiveness; ⑩ Visual analogue scale (VAS).

CR = conventional rehabilitation, ES = electrical stimulation, MM = myofascial manipulation, MS = magnetic stimulation, ST = strength training.

### 3.2. Risk assessment

**Figure 2.**
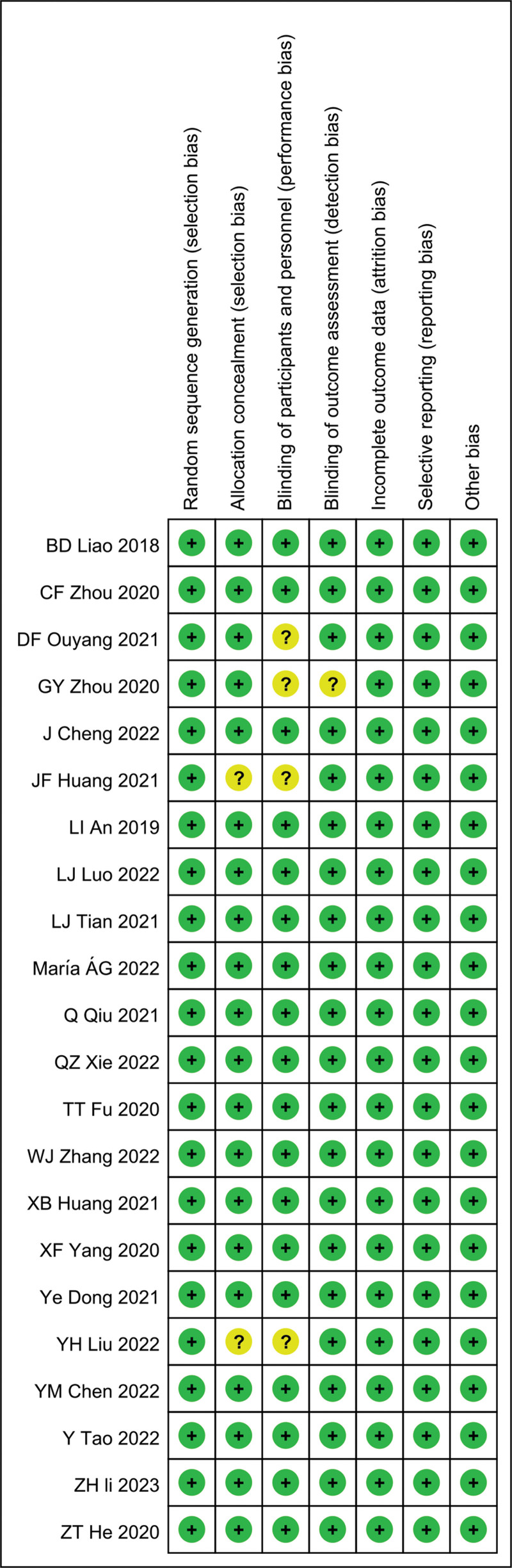
Risk assessment.

Cochrane risk assessment of bias showed insufficient randomization, lack of performance bias in 4 articles, selective bias in 2 articles, lack of detection bias in 1 article, and low risk levels in the remaining articles. Differences between the 2 reviewers were successfully resolved by the third reviewer and consensus was reached (Fig. 2).

### 3.3. Meta-analysis results

#### 3.3.1. Abdominal circumference and rectus abdominis separation distance

**Figure 3.**
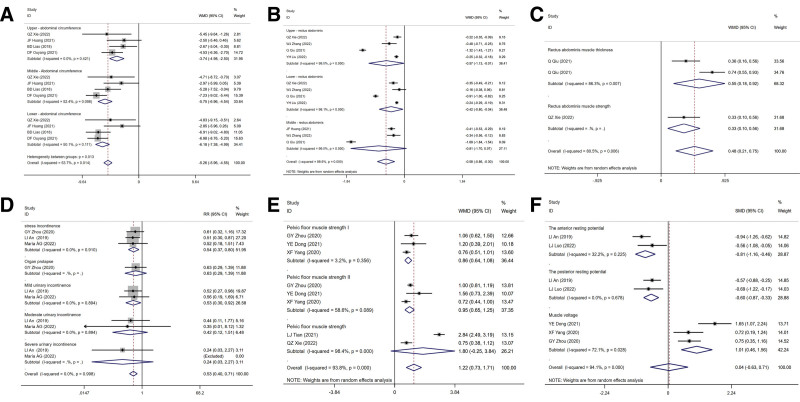
The effect of myofascial manipulation on abdominis problem and pelvic floor problem. (A) Effect of myofascial manipulation on abdominal circumference. (B) Effect of myofascial manipulation on rectus separation index. (C) Effect of myofascial manipulation on rectus abdominis muscle strength and thickness. (D) Effect of myofascial manipulation on pelvic floor function. (E) Effect of myofascial manipulation on pelvic floor muscle strength. (F) Effect of myofascial manipulation on pelvic floor muscle potential.

The result showed that in the myofascial therapy group, abdominal circumference was significantly reduced (*I^2^* = 53.7%, WMD = −5.26, 95% CI [−5.96, −4.55]) (Fig. 3A), as well as the abdominis rectus separation index significantly reduced (*I^2^* = 98.6%, WMD = −0.58, 95% CI [−0.86, −0.30]) (Fig. 3B). Q Qu’s study^[23]^ also reported the thickness change of the rectus abdominis after 1 and 3 months after myofascial therapy, and the thickness of rectus abdominis increased after treatment (*I^2^* = 86.3%, WMD = 0.55, 95% CI [0.18, 0.92]) (Fig. 3C). QZ Xie^[18]^ reported that patients rectus abdominis muscle strength increased after treatment (Fig. 3C).

#### 3.3.2. Pelvic floor muscle function

The prevalence of stress urine incontinence, the incidence of pelvic organ prolapses, the strength of the pelvic floor muscle, and the electromyographic value of the pelvic floor muscle were the key metrics utilized to assess pelvic floor function.

The results showed that the person numbers with various types of pelvic floor muscle dysfunction decreased after myofascial therapy (*I^2^* = 0.0%, WMD = 0.53, 95% CI [0.40, 0.71]) (Fig. 3D). Class I and Class II pelvic floor fibers as well as overall muscle strength significantly enhanced compared to the control group (*I^2^* = 93.8%, WMD = 1.22, 95% CI [0.73, 1.71]) (Fig. 3E). The contraction potential was further divided into 3 subgroups: muscle voltage, pre- and post-resting potential. The results showed that In the myofascial therapy group, both the pre-resting potentials (*I^2^* = 0.0%, WMD = −0.81, 95% CI [−1.16, −0.46]) and post-resting potentials (*I^2^* = 0.0%, WMD = −0.60, 95% CI [−0.87, −0.33]) significantly decreased, while the pelvic floor muscle tension value significantly increased (*I^2^* = 72.1%, WMD = 1.01, 95% CI [0.46, 1.56]) (Fig. 3F).

#### 3.3.3. Pelvic function

**Figure 4.**
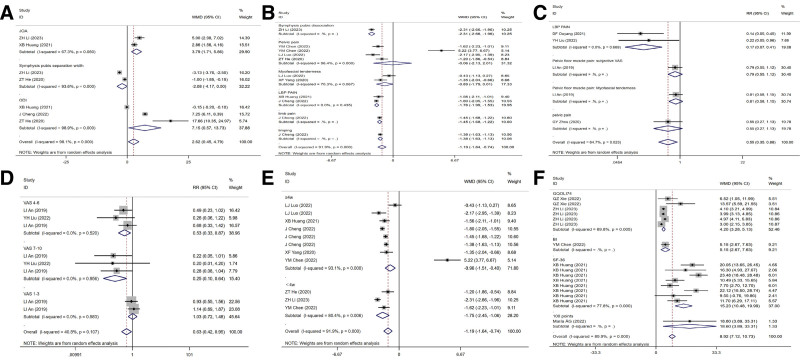
The effect of myofascial manipulation on pelvic problem and pain. (A) Effect of myofascial manipulation on pelvic and pubic symphysis dissociation. (B) Effect of myofascial manipulation for different types of pain (continuous variable). (C) Effect of myofascial manipulation for different types of pain (binary variable). (D) Effect of myofascial manipulation for different pain levels (binary variable). (E) Effect of myofascial manipulation on pain relief at different treatment durations (continuous variable). (F) Effect of myofascial manipulation on ADL. ADL = activity of daily living.

The JOA and ODI measures, along with lumbar radiographs were used to determine the pubic symphysis’ separation distance for evaluating pelvic function. The results showed that the ODI score (*I^2^* = 98.9%, WMD = 7.15, 95% CI [0.57, 13.73]) reduced and the JOA score (*I^2^* = 67.3%, WMD = 3.79, 95% CI [1.71, 5.86]) increased in myofascial therapy group. Lumbar radiographs also revealed that after myofascial therapy, the separation distance of the pubic symphysis was greatly reduced (*I^2^* = 93.6%, WMD = −2.08, 95% CI [−4.17, 0.00]) (Fig. 4A).

#### 3.3.4. Pain

Based on the collected data, this study analyzed the VAS pain score for binary variables and continuous variables, respectively. Continuous variable results showed that myofascia therapy alleviated pain caused by symphysis pubis separation, pelvic pain, limb pain, and claudication (*I^2^* = 91.9%, WMD = −1.19, 95% CI [−1.64, −0.74]) (Fig. 4B). Dichotomous variable results showed that postpartum lumbago, subjective pelvic floor pain, and pelvic floor tenderness decreased after myofascial therapy (*I^2^* = 72.4%, WMD = 0.53, 95% CI [0.30, 0.94]) (Fig. 4C). Subgroup analysis by pain level showed significant pain reduction after myofascial therapy (*I^2^* = 40.8%, WMD = 0.63, 95% CI [0.42, 0.95]) (Fig. 4D). Subgroup analysis for different duration of treatment showed that pain score decreased when treatment < 4 weeks (*I^2^* = 80.4%, WMD = −1.75, 95% CI [−2.45, −1.06]) compared with that when treatment ≥ 4 weeks (*I^2^* = 93.1%, WMD = −0.96, 95% CI [−1.51, −0.40]) more (Fig. 4E).

#### 3.3.5. Ability to perform ADL

Five literatures reported the changes in patients’ daily life activities, and different daily life activities were classified and discussed. Subgroup analysis of different scales of daily living activity was performed in this study, and the results showed that compared with the control group, ADL scores were significantly improved after myofascia therapy (*I^2^* = 89.9%, WMD = 8.92, 95% CI [7.12, 10.73]) (Fig. 4F).

#### 3.3.6. Total effective rate

**Figure 5.**
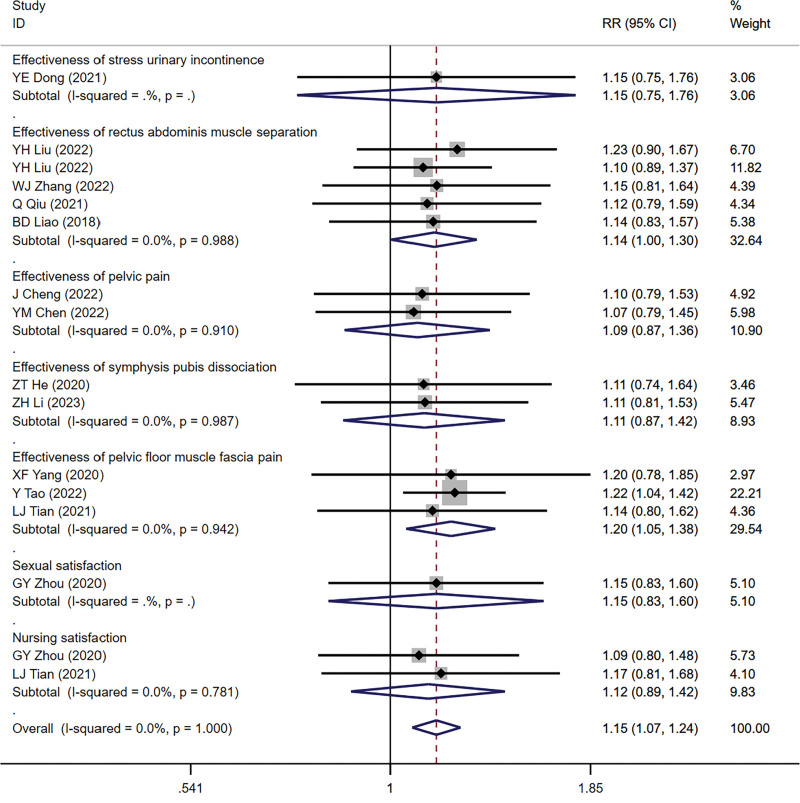
Effect of myofascial manipulation on different postpartum dysfunction.

When the patient’s symptoms showed no obvious improvement or even aggravation after treatment, ineffective was recorded. It is marked as effective when the patient’s symptoms improve or disappear completely. Comparison of treatment effects between the 2 groups showed that after myofascial therapy, the number of patients with stress incontinence was significantly reduced (WMD = 1.15, 95% CI [0.75, 1.76]), while the number with improved rectus abdominis separation symptom (*I^2^* = 0.0%, WMD = 1.14, 95% CI [1.00, 1.3]), pelvic pain relief (*I^2^* = 0.0%, WMD = 1.09, 95% CI [0.87, 1.36]), symphysis pubis separation improvement (*I^2^* = 0.0%, WMD = 1.11, 95% CI [0.87, 1.42]), pelvic myofascial pain relief (*I^2^* = 0.0%, WMD = 1.2, 95% CI [1.05, 1.38]), sexual satisfaction (WMD = 1.15, 95% CI [0.83, 1.6]), and nursing satisfaction (*I^2^* = 0.0%, WMD = 1.12, 95% CI [0.89, 1.42]) was significantly increased (Fig. 5).

### 3.4. The grade

We used Gradeprofiler 3.6 to evaluate the level of evidence for outcome indicators. We assessed “risk of bias,” “inconsistency,” “indirectness,” “imprecision,” and “other considerations” to determine the quality level of the results. Abdominal circumference, rectus abdominalis separation index, pain and treatment effectiveness were all of high qualities evidence. The evidence quality of JOA, ODI, electromyography of pelvic floor, VAS (Rectus abdominis muscle separation and Pelvic floor function), and ADL was low, and the recommendation strength was weak. (Table 2. GRADE).

**Table 2:** GRADE.

Outcomes	Risk of bias	Inconsistency	Indirectness	Imprecision	Other considerations	Overall certainty of evidence
Abdominal circumference	Not serious	Not serious	Not serious	Not serious	Strong correlation	⊕⊕⊕⊕High
Rectus abdominis	Not serious	Not serious	Not serious	Not serious	Strong correlation	⊕⊕⊕⊕High
VAS	Not serious	Serious	Not serious	Not serious	Strong correlation	⊕⊕⊕⊕High
JOA	Not serious	Serious	Not serious	Not serious	None	⊕⊕Low
ODI	Not serious	Serious	Not serious	Not serious	None	⊕⊕Low
ADL	Not serious	Not serious	Not serious	Not serious	None	⊕⊕⊕Moderate
Electromyography	Not serious	Serious	Not serious	Not serious	None	⊕⊕Low
Therapeutic effectiveness	Not serious	Not serious	Serious	Not serious	Strong correlation	⊕⊕⊕⊕High

ADL = activity of daily living, JOA = Japanese Orthopaedic Association scores, ODI = Oswestry disability index, VAS = visual analogue scale.

## 4. Discussion

### 4.1. Research results

Although myofascial therapy was widely used in musculoskeletal disorders, there was little evidence to support the treatment of postpartum problems. This study was the first to analyze the effects of myofascial therapy on postpartum rectus abdominis separation, pelvic floor muscle dysfunction, and lumbago through meta-analysis.

#### 4.1.1. Rectus abdominis muscle separation

Our meta-analysis showed that after myofascial therapy, the abdominal circumference of the patients was significantly reduced, the separation distance of the rectus abdominis was shortened, the thickness of the rectus abdominis was increased and the muscle strength of the rectus abdominis was increased, suggesting that after myofascial therapy, the rectus abdominis was restored to the correct biomechanical position and the elasticity of the rectus abdominis was increased.

#### 4.1.2. Pelvic floor muscle function

Subgroup analysis showed that the number of patients with stress urinary incontinence and pelvic organ prolapse decreased significantly after myofascial therapy. The results of the freehand muscle strength test/pelvic pressure test showed that myofascial therapy was effective in increasing the strength of patients’ pelvic floor muscles. According to Glazer assessment criteria, the anterior and posterior resting stages were normal 2 to 4 μV, and > 4 μV indicated excessive pelvic floor muscle activity. Electromyography showed a decrease in excessive pelvic floor muscle activity in the anterior and posterior resting stages.

#### 4.1.3. Pelvic function

In this study, a meta-analysis was conducted on the status of patients’ lumbar function. The JOA scale selected mainly reflected patients lumbar function. The lower the score, the more obvious the dysfunction. The ODI scale shows the impact of the patient’s lower back pain on daily life, and the higher the score, the more significant the dysfunction. The results showed significant improvement in lumbar function after myofascial therapy and a significant reduction in the separation distance of the symphysis pubis on radiographs, suggesting that myofascial therapy effectively helped patients regain the correct biomechanical position.

#### 4.1.4. Pain

In this study, pain indicators were analyzed by dichotomous and continuous variables, respectively. The results showed that after myofascial therapy, various types of pain were significantly relieved, and the number of pain patients decreased, suggesting that myofascial therapy can reduce the sensitivity of myofascial patients.

#### 4.1.5. ADL and total effective rate

This study showed that when the symptoms of rectus abdominis muscle separation, pelvic floor muscle dysfunction or pelvic dysfunction symptoms were relieved, the patients ADL ability was also enhanced, and sexual satisfaction and nursing satisfaction were improved.

### 4.2. Analysis mechanism

#### 4.2.1. Rectus abdominis muscle separation

The main reason for the separation of rectus abdominis is that the abdominal muscles of pregnant women are elongated and present a state of high tension, and the fascia between the rectus abdominis on both sides becomes thinner due to stretching and gradually separates.^[37]^ Decompensated relaxation of long-term elongated abdominal muscles, coupled with increased hormones during pregnancy and childbirth, such as progesterone, relaxin, and estrogen, can relax soft tissues. It shows decreased muscle strength, decreased fascia elasticity and decompensation, resulting in separation of rectus abdominis and decreased body core stability. Restoring the elasticity of the myofascial membrane helps shorten the separated rectus abdominis. In this study, it was discovered that after receiving myofascial therapy, patients’ rectus abdominis thickness and muscular strength increased and their separation index of abdominal circumference and rectus abdominis dramatically decreased. Therefore, the main principle of myofascial therapy is to increase rectus abdominis muscular strength, reduce rectus abdominis stress, and restore myofascial suppleness.

#### 4.2.2. Pelvic floor muscle function

Pelvic floor muscles and bone and fascia components work together to preserve the integrity of pelvic floor function.^[38]^ The gradual increase of the pressure on the pelvic floor muscle in pregnant women and the excessive stretch, the damage of the pelvic floor muscle fascia during childbirth, the inflammatory factors generated in the process of postpartum pelvic floor tissue repair, and pelvic instability can all lead to postpartum pelvic floor dysfunction, and the most common symptoms are stress incontinence and pelvic organ prolapse. According to the study’s findings, myofascial therapy could significantly reduce patients’ symptoms of stress urinary incontinence and pelvic organ prolapse, increasing the pelvic floor muscle’s potential risk. Myofascial therapy might be able to increase the elasticity of the pelvic floor muscle fascia and increase the contractility of the muscle. Postpartum patients with pelvic floor dysfunction due to pain and vaginal relaxation often show indifference to the sex life, even rejection, seriously affecting the relationship between husband, and wife. Fascia manipulation, acting on myofascial pain and its related points, can effectively relieve postpartum pelvic fascia pain, increase the contraction ability of the muscles involved, and help improve sexual satisfaction.

#### 4.2.3. Pelvic function and pain

Pain is a major postpartum problem, often in the lower back and pelvic floor muscles surrounding the tissue. Histological studies have shown the presence of pain-sensing nerve endings in the lumbago fascia. which often manifest as ischemia and inflammation as well as thickening of the lumbago and dorsal muscle fasciain patients with chronic lumbago,^[39]^ acting as pain triggers. Studies have shown that low back pain can stimulate muscles in the post-motor myofascial chain, resulting in reduced spinal stability, leading to forward pelvis, and further aggravating low back pain.^[40]^ Because of the long-term load, pelvic floor muscle fascia and muscle fiber contraction and even spasm, compression of the surrounding nerve tissue, also easy to form local pain trigger points. Activation of myofascial pain trigger points leads to dilation or even dislocation of the symphysis pubis space.^[41]^Postpartum symphysis pubis reduction may also be hindered by local or referred pain. In this study, we analyzed pain from a variety of angles, the results showed that with myofascial therapy, the patients’ lumbago and pelvic floor myofascial pain symptoms were significantly reduced, and separation distance was significantly shortened. It was suggested that myofascial therapy may inactivate the trigger points of myofascial pain. On the other hand, it could promote the human body to restore the correct biomechanical position and reduce the pain caused by mechanical changes.

#### 4.2.4. Others

This study conducted a meta-analysis of daily living capacity activities to assess patients quality of life, including physiological function, physiological function, physical pain, general health status, energy, mental health daily activities, sleep, etc. It was found that when patients’ rectus abdominis separation, lumbago, pubic symphysis separation, and pelvic floor muscle dysfunction symptoms improved, their ability to perform daily activities also improved.

This study confirmed that myofascial therapy can reduce the tension of abnormal myofascial membrane, promote the recovery of physiological elasticity and strength of fascia and muscle tissue, and restore the correct and normal biomechanics of the body, so as to reduce the symptoms of postpartum rectus separation, pelvic floor muscle dysfunction and back pain caused by myofascial dysfunction. Other postpartum disorders such as puerperal cervical spondylosis, stenosing tendovaginitis radial styloid, flat feet, and heel pain are also associated with myofascial system disorders, and therefore, these disorders may also be treated with myofascial therapy.

### 4.3. Limitation

Postpartum dysfunction includes rectus abdominis dissociation, pelvic leaning forward, lumbago, sacroiliac joint disorders, sacral pain, pelvic floor muscle dysfunction, and stenosing tendovaginitis radial styloid. It was found that the clinical evaluation and treatment data of postpartum dysfunction were mainly in the aspects of rectus abdominis separation, pain and pelvic floor dysfunction, and other studies were few. At present, there is a lack of systematic evaluation of postpartum dysfunction. In the future, it is necessary to design a set of comprehensive evaluation methods for women’s postpartum dysfunction. In treatment, there are few studies on myofascial therapy, lack of efficacy comparison with other treatment methods, and long-term efficacy has not been reported. In the future, better randomized controlled trials with larger sample sizes and multi-centers should be designed to conduct prospective, randomized controlled trials to explore the short-term and long-term efficacy of myofascial therapy in the treatment of postpartum women with various functional disorders.

## 5. Conclusion

In summary, myofascial therapy could reduce abdominal circumference, relieve the pain of lumbar and pelvic floor muscle fascia, improve the contractile strength of pelvic floor muscle, improve the ability of daily living activities, and play a positive role in improving postpartum health, maintaining husband and wife life, and improving the relationship between husband and wife. It is worth applying in clinic.

## Acknowledgments

Thanks to the Education Department of Jilin Province for providing financial support for this study. Thanks to Lijing Zhao, Lirong Guo and Bing Liang from the School of Nursing, Jilin University for their contributions to this article.

## Author contributions

**Conceptualization:** Shuang Zhang.

**Data curation:** Peiqiang Peng, Hong Liu.

**Formal analysis:** Yueting Wang, Peiqiang Peng.

**Funding acquisition:** Wenxi He, Shuang Zhang.

**Investigation:** Yueting Wang, Peiqiang Peng, Hong Liu.

**Methodology:** Haitao Zhang, Haiyan Xu.

**Project administration:** Wenxi He, Haitao Zhang.

**Resources:** Yueting Wang, Peiqiang Peng.

**Software:** Wenxi He, Haitao Zhang.

**Supervision:** Shuang Zhang.

**Validation:** Shuang Zhang.

**Visualization:** Haitao Zhang, Haiyan Xu, Hong Liu.

**Writing – original draft:** Yueting Wang, Hong Liu.

**Writing – review & editing:** Yueting Wang, Peiqiang Peng.

## Supplementary Material
