# Supplementary material for: The effect of myofascial therapy on postpartum rectus abdominis separation, low back and leg pain, pelvic floor dysfunction: A systematic review and meta-analysis
Source: Medicine (Baltimore). 2023 Nov 3;102(44):e35761. doi: 10.1097/MD.0000000000035761 (PMC10627697; doi:10.1097/MD.0000000000035761)
Supplement: Supplementary file 1 [file medi-102-e35761-s001.docx]

**Supplement Table 1. Patients' basic information**

| Study | year | Number | | Patients’ years | | Number of pregnancies | | Female delivery mode | | gestation time | | postpartum day | |
| --- | --- | --- | --- | --- | --- | --- | --- | --- | --- | --- | --- | --- | --- |
|  |  | E | C | E | C | E | C | E | C | E | C | E | C |
|  |  |  |  |  |  | Single/twin | Single/twin | eutocia/cesarean | eutocia/cesarean |  |  |  |  |
| QZ Xie | 2022 | 31 | 31 | 30.18±5.72 | 29.84±5.47 | 20/11 | 21/10 | none | none | 38.14±1.44 | 38.02±1.36 | none | none |
| JF Huang | 2021 | 48 | 48 | 29.23±2.62 | 28.75±2.43 | 45/6 | 46/5 | 32/16 | 31/17 | 6w | 6w | 6w | 6w |
| GY Zhou | 2020 | 50 | 50 | 27.5±3.55 | 27.48±3.59 | none | none | 50/0 | 50/0 | none | none | none | none |
| WJ Zhang | 2022 | 40 | 40 | 29±1.4 | 28±1.5 | none | none | none | none | none | none | none | none |
| TT Fu | 2020 | 35 | 35 | 30.3±7.09 | 29.66±7.28 | 1.26±0.53 | 1.31±0.5 | 17/18 | 19/16 | 5.92m±3.19 | 6.24m±3.47 | 5.92m±3.19 | 6.24m±3.47 |
| ZH Li | 2023 | 51 | 51 | 26.03±5.12 | 25.12±5.17 | 37/14 | 36/15 | none | none | 39.29±5.08 | 39.27±5.06 | none | none |
| Q Qiu | 2021 | 38 | 38 | 27.59±4.38 | 27.81±4.52 | none | none | 24/14 | 25/13 | none | none | none | none |
| Y Tao | 2022 | 225 | 224 | 42.51±5.2 | 42.58±5.13 | 1.76±0.3 | 1.81±0.31 | none | none | none | none | none | none |
| BD Liao | 2018 | 50 | 50 | 29.36±2.68 | 29.46±2.73 | 37/13 | 38/12 | 34/16 | 35/15 | none | none | none | none |
| LJ Tian | 2021 | 40 | 40 | 25.78±4.01 | 24.55±3.58 | 1.42±0.36 | 1.21±0.51 | none | none | none | none | none | none |
| YM Chen | 2022 | 50 | 50 | 29.27±5.13 | 29.53±5.11 | 31/19 | 32/18 | none | none | none | None | none | none |
| LI An | 2019 | 100 | 70 | 18-40 | 18-40 | 100/0 | 70/0 | none | none | none | None | 42 | 42 |
| DF Ouyang | 2021 | 65 | 65 | 26.33±3.13 | 26.41±3.05 | none | none | none | none | 38.5±0.7 | 38.44±0.65 | none | none |
| Ye Dong | 2021 | 30 | 30 | 28.24±3.84 | 28.24±3.84 | none | none | 25/5 | 23/7 | none | none | none | none |
| CF Zhou | 2020 | 102 | 0 | 20-40（29.5） | - | 102/0 | - | 66/36 | - | none | - | none | - |
| LJ Luo | 2022 | 30 | 30 | 29.27±4.29 | 30.57±4.2 | 1.47±0.57 | 1.37±0.55 | 11月19日 | 10月20日 | none | none | none | none |
| YH Liu | 2022 | 100 | 100 | 27.6±3.9 | 27.5±3.8 | 1.8±0.6 | 1.7±0.6 | 30/70 | 29/71 | none | none | 15.3±3 | 15.5±3.1 |
| XB Huang | 2021 | 100 | 100 | 32.25±4.39 | 32.18±5.48 | none | none | none | none | 38.97±1.42 | 38.55±1.7 | none | none |
| J Cheng | 2022 | 42 | 44 | 29.34±3.19 | 28.14±3.12 | none | none | none | none | 37.32±2.61 | 37.18±2.59 | none | none |
| ZT He | 2020 | 30 | 30 | 27.23±2.22 | 26.43±2.96 | none | none | none | none | none | none | none | none |
| XF Yang | 2020 | 29 | 29 | 30.41±4.04 | 29.34±4.52 | 2.76±0.95 | 2.34±0.86 | 19/10 | 20/9 | 39.04±1.11 | 38.93±1.14 | none | none |
| María ÁG | 2022 | 27 | 27 | 33.8±3.8 | 30.7±4.3 | 1.4±0.6 | 1.4±0.5 | none | none | 39.5±1.2 | 39±2.3 | none | none |
