# Supplementary material for: The effect of myofascial therapy on postpartum rectus abdominis separation, low back and leg pain, pelvic floor dysfunction: A systematic review and meta-analysis
Source: Medicine (Baltimore). 2023 Nov 3;102(44):e35761. doi: 10.1097/MD.0000000000035761 (PMC10627697; doi:10.1097/MD.0000000000035761)
Supplement: Supplementary file 2 [file medi-102-e35761-s002.pdf]

Chinese MESH:

肌筋膜手法、肌筋膜激痛点、推拿手法、CC 点、脏器筋膜手法

骨盆：骨盆错位、耻骨联合分离、下背痛、腰痛、腰椎失稳、骨盆前倾、骶髂关节紊乱、尾骨痛、尾骨错位、骶髂关节痛、

腹直肌：腹直肌分离、腰围增大、腹部皮下脂肪增厚

盆底肌：盆腔器官脱垂、性功能障碍、尿失禁、慢性盆腔疼痛、盆底肌功能障碍、便失禁、便秘、排尿困难、盆底肌无力

女性产后

CNKI：---

肌筋膜手法、肌筋膜激痛点、推拿手法、CC 点、脏器筋膜手法　　7751

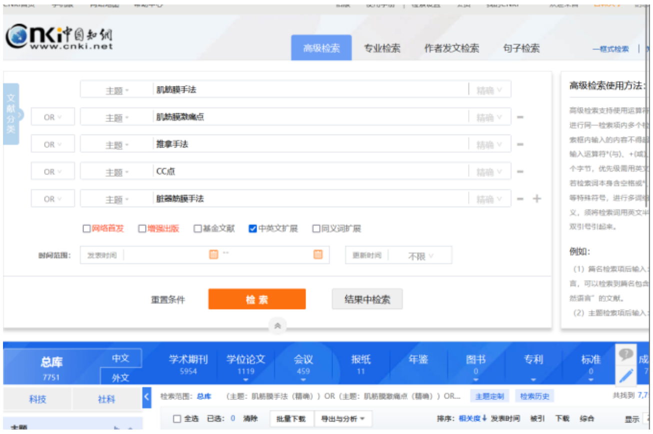

骨盆：骨盆错位、耻骨联合分离、下背痛、腰痛、腰椎失稳、骨盆前倾、骶髂关节紊乱、尾骨痛、尾骨错位、骶髂关节痛　　291

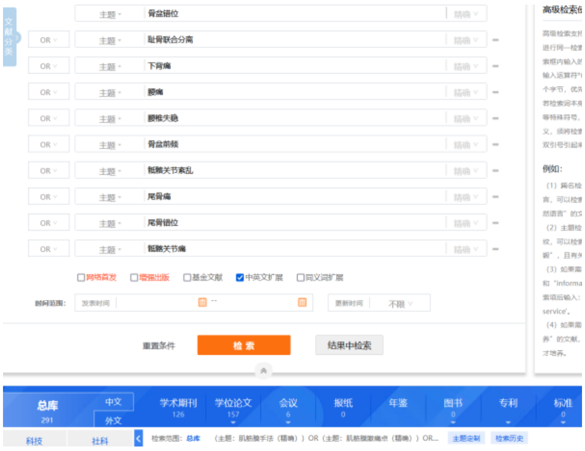

腹直肌：腹直肌分离、腰围增大、腹部皮下脂肪增厚　　2

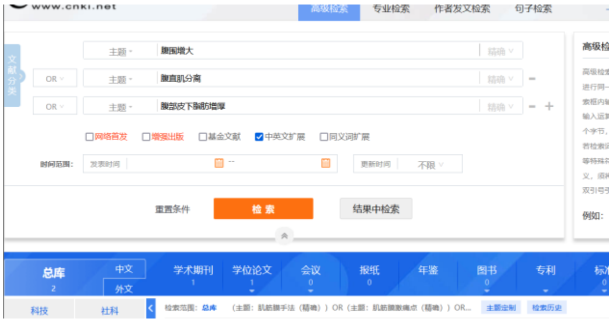

盆底肌：盆腔器官脱垂、性功能障碍、尿失禁、慢性盆腔疼痛、盆底肌功能障碍、便失禁、便秘、排尿困难、盆底肌无力　　93

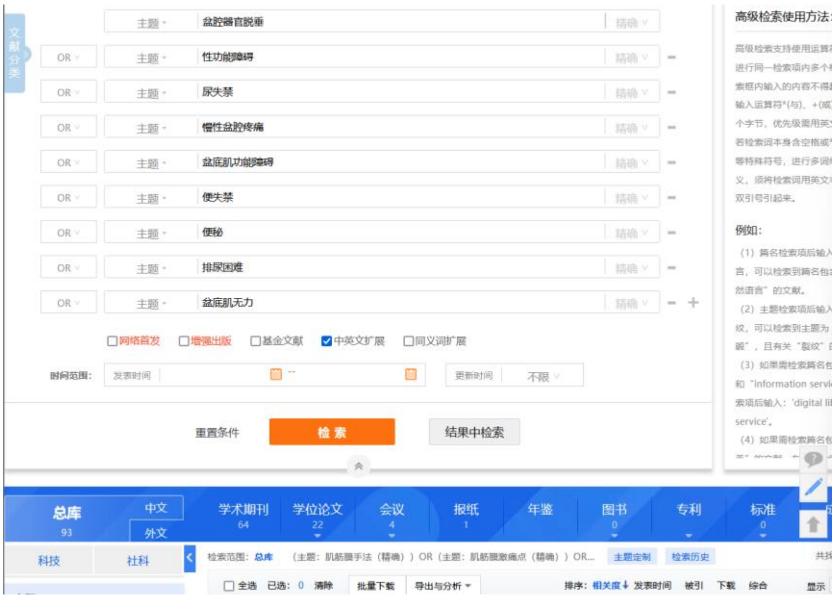

女性产后

Wanfang

筋膜：44247

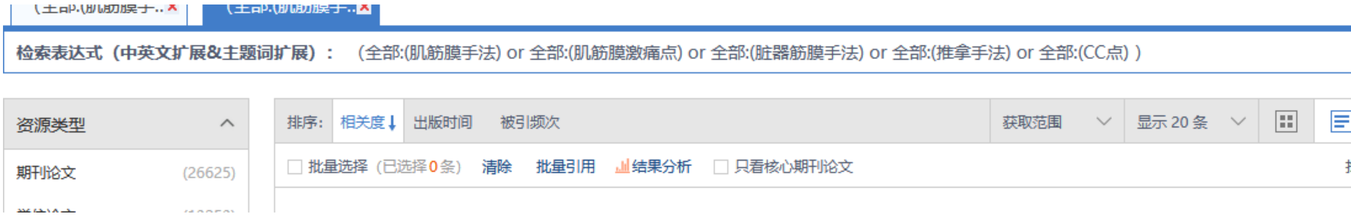

MESH：（全部:(肌筋膜手法) or 全部:(肌筋膜激痛点) or 全部:(脏器筋膜手法) or 全部:(推拿手法) or 全部:(CC 点) ）and (全部:(骨盆错位) or 全部:(耻骨联合分离)

or 全部:(下背痛) or 全部:(腰痛) or 全部:(腰椎失稳) or 全部:(骨盆前倾) or 全部:(骶髂关节紊乱) or 全部:(尾骨痛) or 全部:(尾骨错位) or 全部:(骶髂关节痛))

982

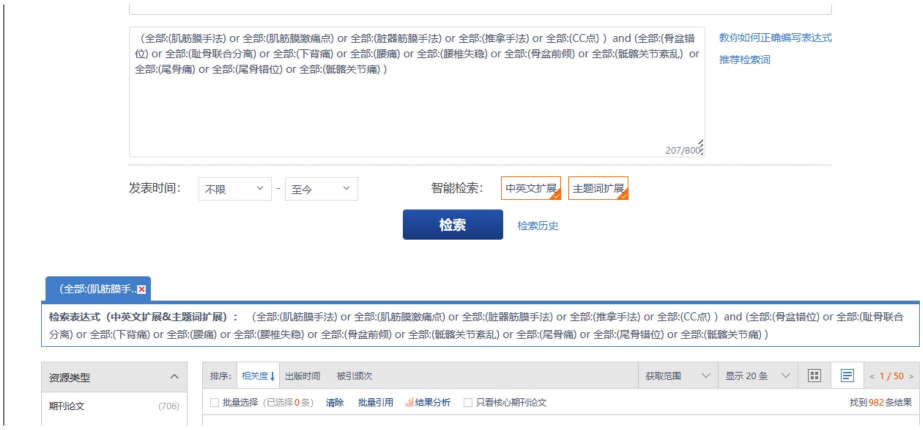

（全部:(肌筋膜手法) or 全部:(肌筋膜激痛点) or 全部:(脏器筋膜手法) or 全部:(推拿手法) or 全部:(CC 点) ） and (全部:(腹直肌分离) or 全部:(腰围增大) or 全部:(腹部皮下脂肪增厚))

16

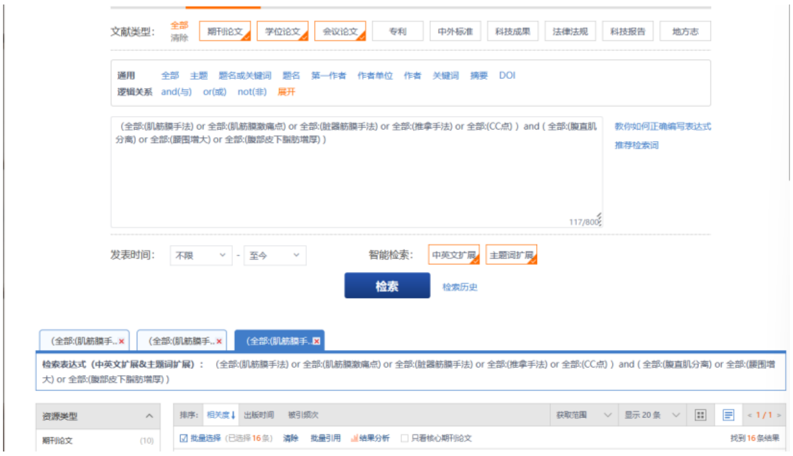

（全部:(肌筋膜手法) or 全部:(肌筋膜激痛点) or 全部:(脏器筋膜手法) or 全部:(推拿手法) or 全部:(CC 点) ） and (全部:(盆腔器官脱垂) or 全部:(性功能障碍) or 全部:(尿失禁) or 全部:(慢性盆腔疼痛) or 全部:(盆底肌功能障碍) or 全部:(便失禁) or 全部:(便秘) or 全部:(排尿困难) or 全部:(盆底肌无力))

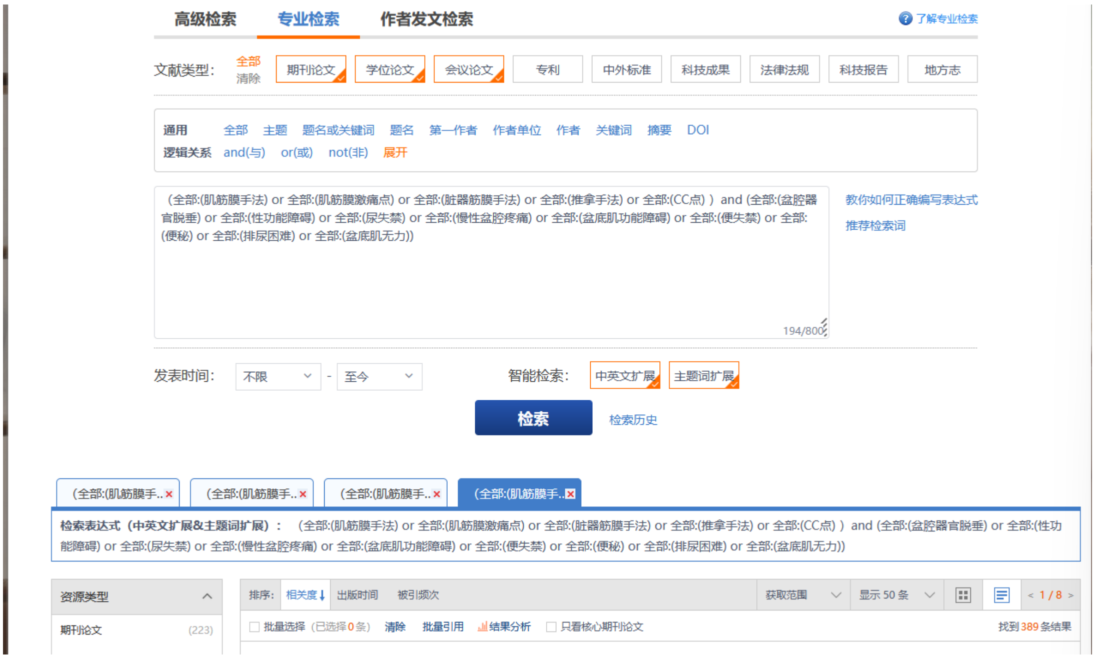

VIP：

MESH：((((任意字段=肌筋膜手法 OR 任意字段=肌筋膜激痛点) OR 任意字段=CC 点) OR 任意字段=推拿手法) OR 任意字段=脏器筋膜手法)

肌筋膜手法、肌筋膜激痛点、推拿手法、CC 点、脏器筋膜手法　　11205

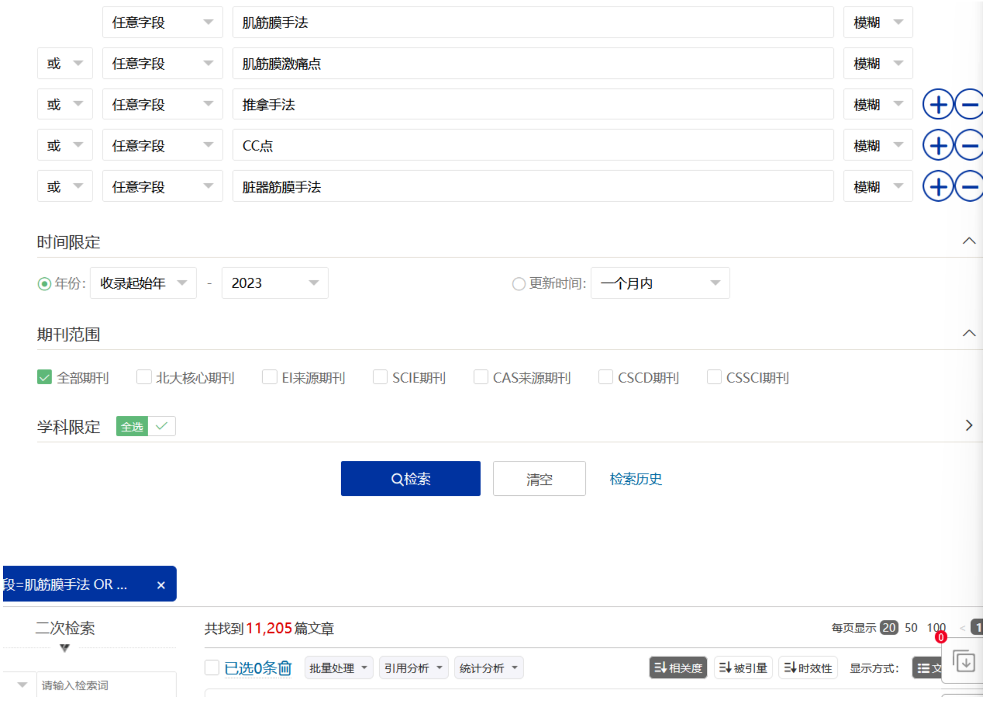

骨盆：骨盆错位、耻骨联合分离、下背痛、腰椎失稳、骨盆前倾、腰痛、骶髂关

节紊乱、尾骨痛、尾骨错位、骶髂关节痛　235

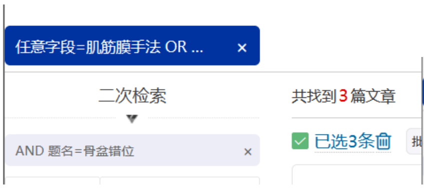

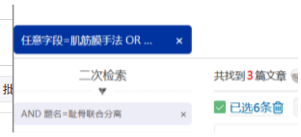

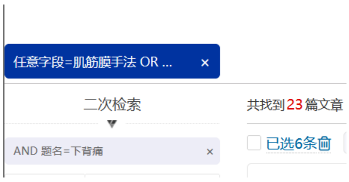

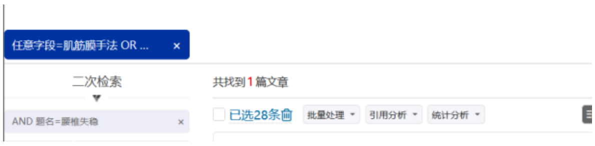

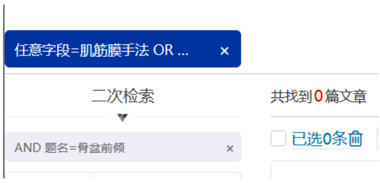

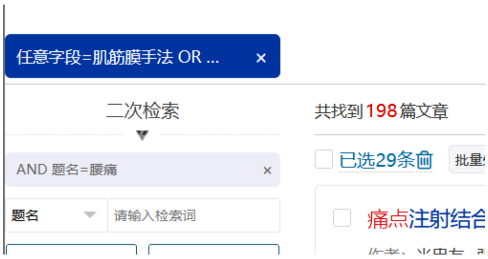

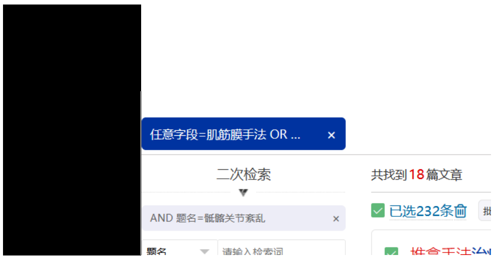

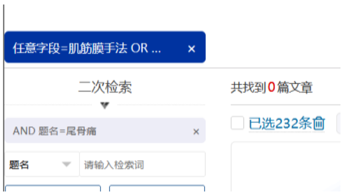

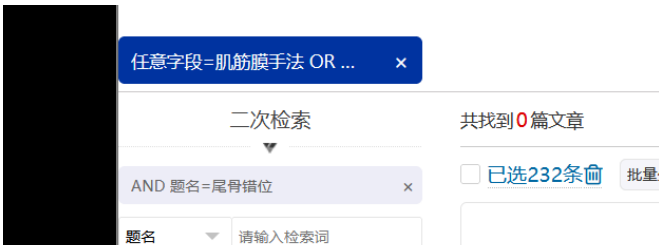

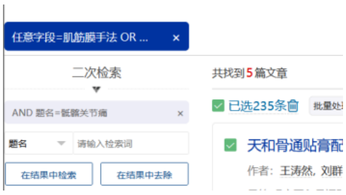

腹直肌：腹直肌分离、腰围增大、腹部皮下脂肪增厚　6

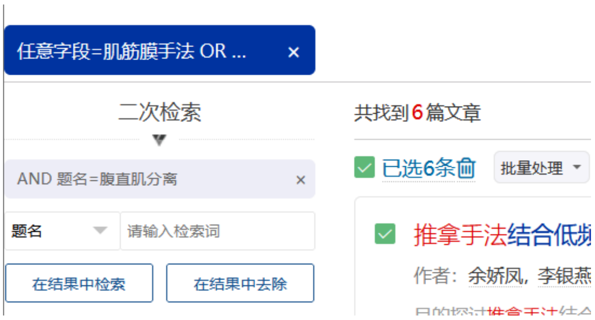

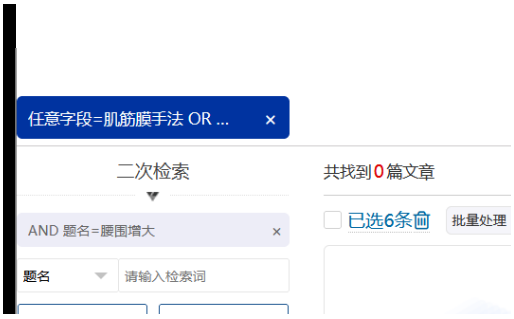

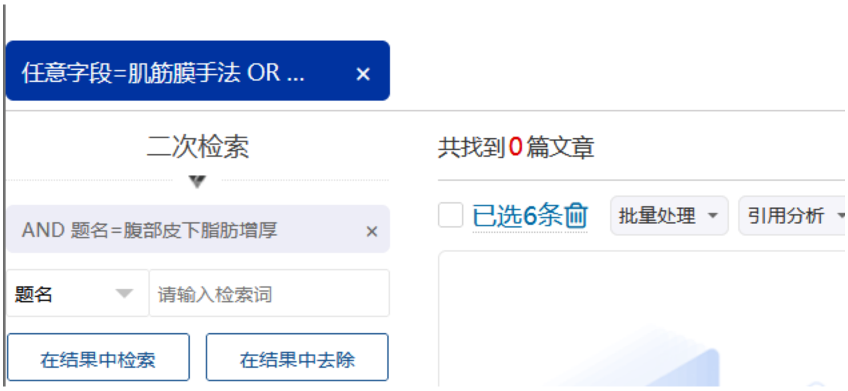

盆底肌：盆腔器官脱垂、性功能障碍、尿失禁、慢性盆腔疼痛、盆底肌功能障碍、便失禁、便秘、排尿困难、盆底肌无力

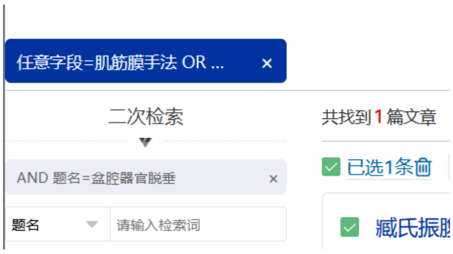

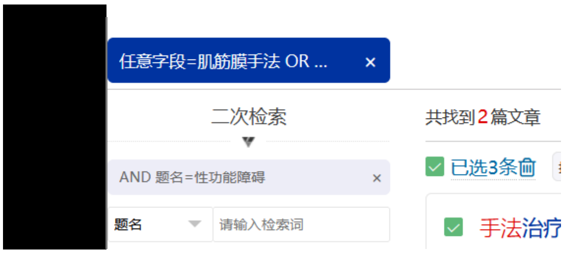

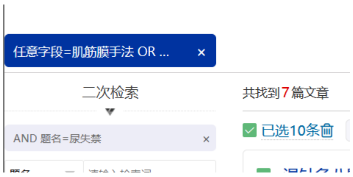

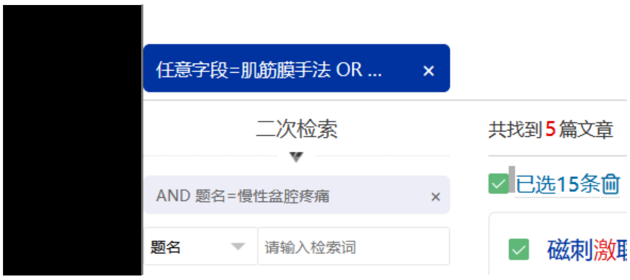

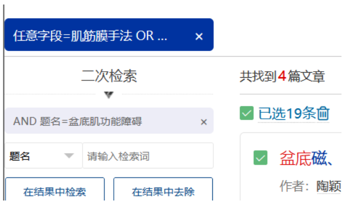

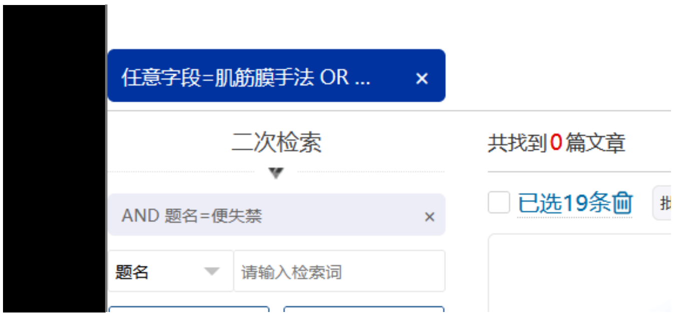

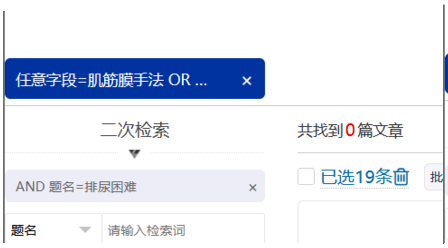

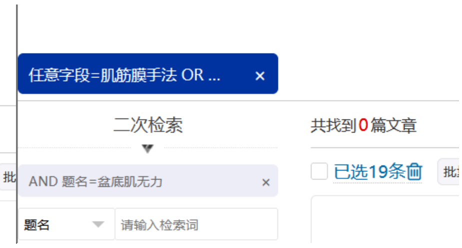

ENGLISH MESH:

Myofascial manipulation, myofascial pain point therapy, Massage manipulation, CC point, fascial manipulation of viscera

Pelvic dislocation, pubic symphysis separation, lower back pain, non-specific low back pain, lumbago, lumbar instability, pelvic forward, sacroiliac joint disorder, tailbone pain, tailbone dislocation, sacroiliac joint pain

Rectus abdominis muscle separation, waist circumference enlargement, abdominal subcutaneous fat thickening

Pelvic misalignment, sexual dysfunction, urinary incontinence, chronic pelvic pain, pelvic floor muscle dysfunction, fecal incontinence, constipation, dysuria, pelvic floor muscle weakness

postpartum

Pubmed

((((Myofascial manipulation) OR (myofascial pain point therapy)) OR (Massage manipulation)) OR (CC point)) OR (viscera fascial manipulation)

(((((((((Pelvic dislocation) OR (pubic symphysis separation)) OR (lower back pain)) OR (non-specific low back pain)) OR (lumbago)) OR (lumbar instability)) OR (pelvic forward)) OR (sacroiliac joint disorder)) OR (tail bone pain)) OR (tailbone dislocation)) OR (sacroiliac joint pain)

((Rectus abdominis muscle separation) OR (waist circumference enlargement)) OR (abdominal subcutaneous fat thickening)

((((((((Pelvic misalignment) OR (sexual dysfunction)) OR (urinary incontinence)) OR (chronic pelvic pain)) OR (pelvic floor muscle dysfunction)) OR (fecal incontinence)) OR (constipation)) OR (dysuria)) OR (pelvic floor muscle weakness)

History and Search Details   Download   Delete

Search	Actions	Details	Query	Results	Time
#18	•••	>	Search: (((((Myofascial manipulation) OR (myofascial pain point therapy)) OR (Massage manipulation)) OR (CC point)) OR (viscera fascial manipulation)) AND ((((((((Pelvic misalignment) OR (sexual dysfunction)) OR (urinary incontinence)) OR (chronic pelvic pain)) OR (pelvic floor muscle dysfunction)) OR (fecal incontinence)) OR (constipation)) OR (dysuria)) OR (pelvic floor muscle weakness))	183	09:41:11
#17	•••	>	Search: (((((Myofascial manipulation) OR (myofascial pain point therapy)) OR (Massage manipulation)) OR (CC point)) OR (viscera fascial manipulation)) AND (((Rectus abdominis muscle separation) OR (waist circumference enlargement)) OR (abdominal subcutaneous fat thickening))	1	09:40:42
#16	•••	>	Search: (((((Myofascial manipulation) OR (myofascial pain point therapy)) OR (Massage manipulation)) OR (CC point)) OR (viscera fascial manipulation)) AND ((((((((((Pelvic dislocation) OR (pubic symphysis separation)) OR (lower back pain)) OR (non-specific low back pain)) OR (lumbago)) OR (lumbar instability)) OR (pelvic forward)) OR (sacroiliac joint disorder)) OR (tail bone pain)) OR (tailbone dislocation)) OR (sacroiliac joint pain))	373	09:40:23
#15	•••	>	Search: ((((((((Pelvic misalignment) OR (sexual dysfunction)) OR (urinary incontinence)) OR (chronic pelvic pain)) OR (pelvic floor muscle dysfunction)) OR (fecal incontinence)) OR (constipation)) OR (dysuria)) OR (pelvic floor muscle weakness)	147,747	09:39:46
#14	•••	>	Search: ((Rectus abdominis muscle separation) OR (waist circumference enlargement)) OR (abdominal subcutaneous fat thickening)	697	09:39:35
#13	•••	>	Search: ((((((((((Pelvic dislocation) OR (pubic symphysis separation)) OR (lower back pain)) OR (non-specific low back pain)) OR (lumbago)) OR (lumbar instability)) OR (pelvic forward)) OR (sacroiliac joint disorder)) OR (tail bone pain)) OR (tailbone dislocation)) OR (sacroiliac joint pain)	66,448	09:38:47
#12	•••	>	Search: ((((Myofascial manipulation) OR (myofascial pain point therapy)) OR (Massage manipulation)) OR (CC point)) OR (viscera fascial manipulation)	11,413	09:37:53

History and Search Details   Download   Delete

Search	Actions	Details	Query	Results	Time
#23	•••	>	Search: ((((((Myofascial manipulation) OR (myofascial pain point therapy)) OR (Massage manipulation)) OR (CC point)) OR (viscera fascial manipulation)) AND ((((((((Pelvic misalignment) OR (sexual dysfunction)) OR (urinary incontinence)) OR (chronic pelvic pain)) OR (pelvic floor muscle dysfunction)) OR (fecal incontinence)) OR (constipation)) OR (dysuria)) OR (pelvic floor muscle weakness))) AND (postpartum)	3	09:48:29
#22	•••	>	Search: ((((((Myofascial manipulation) OR (myofascial pain point therapy)) OR (Massage manipulation)) OR (CC point)) OR (viscera fascial manipulation)) AND (((Rectus abdominis muscle separation) OR (waist circumference enlargement)) OR (abdominal subcutaneous fat thickening))) AND (postpartum)	0	09:48:02
#20	•••	>	Search: ((((((Myofascial manipulation) OR (myofascial pain point therapy)) OR (Massage manipulation)) OR (CC point)) OR (viscera fascial manipulation)) AND ((((((((((Pelvic dislocation) OR (pubic symphysis separation)) OR (lower back pain)) OR (non-specific low back pain)) OR (lumbago)) OR (lumbar instability)) OR (pelvic forward)) OR (sacroiliac joint disorder)) OR (tail bone pain)) OR (tailbone dislocation)) OR (sacroiliac joint pain))) AND (postpartum)	2	09:47:16

Web of science

1. ((((TS=(Myofascial manipulation)) OR TS=(myofascial pain point therapy)) OR TS=(Massage manipulation)) OR TS=(CC point)) OR TS=(fascial manipulation of viscera)

2. ((((TS=(Myofascial manipulation)) OR TS=(myofascial pain point therapy)) OR TS=(Massage manipulation)) OR TS=(CC point)) OR TS=(fascial manipulation of viscera)

3. ((((TS=(Myofascial manipulation)) OR TS=(myofascial pain point therapy)) OR TS=(Massage manipulation)) OR TS=(CC point)) OR TS=(fascial manipulation of viscera)

4. ((((((((((TS=(Pelvic dislocation)) OR TS=(pubic symphysis separation)) OR TS=(lower back pain)) OR TS=(non-specific low back pain)) OR TS=(lumbago)) OR TS=(lumbar instability)) OR TS=(pelvic forward)) OR TS=(sacroiliac joint disorder)) OR TS=(tail bone pain)) OR TS=(tailbone dislocation)) OR TS=(sacroiliac joint pain)

8. TS=(postpartum)

11	#7 AND #8	3	添加到检索式
10	#8 AND #6	4	添加到检索式
9	#5 AND #8	0	添加到检索式
8	TS=(postpartum)	112,488	添加到检索式
7	#1 AND #2	315	添加到检索式
6	#1 AND #4	745	添加到检索式
5	#1 AND #3	0	添加到检索式
4	((((((((((TS=(Pelvic dislocation)) OR TS=(pubic symphysis separation)) OR TS=(lower back pain)) OR TS=(non-specific low back pain)) OR TS=(lumbago)) OR TS=(lumbar instability)) OR TS=(pelvic forward)) OR TS=(sacroiliac joint disorder)) OR TS=(tail bone pain)) OR TS=(tailbone dislocation)) OR TS=(sacroiliac joint pain)	100,591	添加到检索式
3	((TS=(Rectus abdominis muscle separation)) OR TS=(waist circumference enlargement)) OR TS=(abdominal subcutaneous fat thickening)	299	添加到检索式
2	((((((((TS=(Pelvic misalignment)) OR TS=(sexual dysfunction)) OR TS=(urinary incontinence)) OR TS=(chronic pelvic pain)) OR TS=(pelvic floor muscle dysfunction)) OR TS=(fecal incontinence)) OR TS=(constipation)) OR TS=(dysuria)) OR TS=(pelvic floor muscle weakness)	186,504	添加到检索式
1	((((TS=(Myofascial manipulation)) OR TS=(myofascial pain point therapy)) OR TS=(Massage manipulation)) OR TS=(CC point)) OR TS=(fascial manipulation of viscera)	20,518	添加到检索式
